# Effect of follicle-stimulating hormone on Bligon goat oocyte maturation and embryonic development post *in vitro* fertilization

**DOI:** 10.14202/vetworld.2020.2443-2446

**Published:** 2020-11-14

**Authors:** Diah Tri Widayati, Mulyoto Pangestu

**Affiliations:** 1 Department of Animal Breeding and Reproduction, Faculty of Animal Science, Universitas Gadjah Mada, Yogyakarta 55281, Indonesia; 2 Department of Animal Reproduction, Faculty of Animal Science, Jenderal Soedirman, Purwokerto, Central Java 53122 Indonesia; 3 Department of Obstetrics and Gynecology, Monash University, Clayton 3168, Australia

**Keywords:** Bligon goat, follicle-stimulating hormone supplementation, *in vitro* embryo production

## Abstract

**Background and Aim:**

Bligon goat is a crossbreed between Etawah and Kacang goat. This crossbreed goat is mostly reared by small farmers. *In vitro* maturation allows female goat (does) contributes toward reproduction despite the fact that the animal has been slaughtered. The aim of this study was to determine the *in vitro* maturation rate of Bligon goat oocytes supplemented with follicle-stimulating hormone (FSH), and their ability for further embryonic development after *in vitro* fertilization.

**Materials and Methods:**

Experiment was conducted at the Laboratory of Animal Physiology and Reproduction, Faculty of Animal Science, Universitas Gadjah Mada, Yogyakarta, using Bligon goat ovaries obtained from local slaughterhouse around Yogyakarta. One thousand five hundred cumulus-oocyte complexes were matured for 24 h in tissue culture medium 199 supplemented with 50 IU/L FSH or without FSH (control). First, matured oocytes were evaluated its morphology based on the expansion of cumulus cells and PB1 extrusion. Next, 600 oocytes were then stained with 1% aceto-orcein to examine maturation based on changes in the configuration of chromosomes and nuclear membrane breakdown. Oocytes were considered mature when they reached metaphase II. To prove the ability of mature oocytes to develop into embryos, 900 oocytes were processed for fertilization *in vitro*. The data were analyzed using analysis of variance.

**Results:**

The results indicated that FSH supplementation significantly increased oocyte maturation rate (65.21±7.26 vs. 43.25±6.23%) as indicated by extrusion of PB1 and homologous chromosome pairing and lined in the equator. The rate of degeneration was lower in the FSH-supplemented medium (3.21±0.25 vs. 10.17±3.15%). The blastocyst stage of oocyte developed embryos was reached by 12.43±2.15% and 22.28±4.86% of the control and treatment groups, respectively.

**Conclusion:**

FSH supplementation significantly improves oocyte maturation and yields mature oocytes for future embryo development *in vitro*.

## Introduction

Bligon goat is a Java island crossbreed between Etawah and Kacang goat (local goat), and their blood composition comprises more than 50% of Kacang [1]. Some of the advantages of this goat include less requirement of land area, high adaptability to the environment, and limited food requirement. Moreover, this animal is also popular for meat production. The high rate of slaughtering of female goats causes an increase in disturbed population. The increase in population of Bligon goat was only 1.94% in 2017 [2]. Therefore, efforts are needed to improve the utilization of slaughtered female goats. The utilization of oocytes collected from the ovaries of abattoir waste extends the use of the female goat post-termination through. Several studies have been carried out using the ovary, which is a byproduct of slaughtered houses, as an effort to save the genetic and reproductive potential of goat through *in vitro* fertilization (IVF) [3-5].

One of the important stages of *in vitro* embryo production is *in vitro* oocytes maturation. The maturity of the oocyte determines the success of the IVF. Numerous studies have been conducted to increase the quantity and quality of the mature oocyte, such as supplementation of the maturation medium with polyvinylpyrrolidone, bovine serum albumin (BSA), calf serum and bovine follicular fluid [6], gonadotropin [3], and insulin growth factor-I [7]. Previous study reported that the presence of follicle-stimulating hormone (FSH) in *in vitro* maturation (IVM) of sow oocytes enhanced nuclear maturation and improved capacity of blastocyst formation [8]. In addition reported that FSH or equine chorionic gonadotropin in maturation medium enhanced cleavage and developmental rates of IVF buffalo embryos [9]. The effects of FSH on oocytes involve the cyclic adenosine 3-5-monophosphate (cAMP) pathway. First, FSH induces cumulus cells to produce cAMP, which is transferred to oocytes through gap junctions. Second, cAMP plays a dual role in the maturation of meiotic oocytes [10]. cAMP mediated of the stimulating active of gonadotropin on the follicle cell, and an inhibiting effect on the oocytes itself [11].

IVM is determined successful if the oocytes reach the metaphase II (MII) stage. This is indicated by cumulus cell expansion and PB1 extrusion. The oocytes are able to reach MII due to internal factors such as quality and genetic viability. This study was conducted to investigate the effect of FSH on Bligon goat oocytes and their ability to reach MII and embryonic development after IVF.

## Materials and Methods

### Ethical approval

No animal ethics approval is required in this research since goat ovaries were scavenged from local slaughterhouse and frozen semen for *in vitro* fertilization were purchased from a commercial source.

### Study period and location

The research was carried out between April and August 2018 at the Laboratory of Animal Physiology and Reproduction, Faculty of Animal Science, Universitas Gadjah Mada, Yogyakarta.

### Material

Goat ovaries were obtained from a local slaughterhouse during routine commercial slaughtering. Frozen Etawah goat semen for *in vitro* fertilization were purchased from Centre for Artificial Insemination, Singosari, East Java. All chemicals and reagents used in this study were produced from Sigma-Aldrich (St. Louis, Mo., USA) unless otherwise stated.

### Oocytes collection and maturation in vitro

The ovaries were removed from the bodies and placed in normal saline solution containing streptomycin, penicillin (Meiji, Indonesia) and transported to the laboratory at 31-34°C within 2 h post slaughtering. Oocyte aspiration was carried with an 18 G needle attached to 3 mL disposable syringe filled with Dulbecco’s phosphate-buffered saline supplemented with 6 mg/mL BSA. Approximately 1000 goat ovaries obtained from 50 collections were used in this experiment. Following follicle aspiration, 4000 cumulus-oocyte complexes (COCs) were collected but only 1500 oocytes were used in this study. COCs with three layers of cumulus cells (Grades A, B, and C) [12] were used in this experiment. The COCs were washed twice in tissue culture medium (TCM)-199 medium supplemented by 2% fetal calf serum (FCS), 100 U/mL penicillin, and 100 μg/mL streptomycin, and different concentrations of FSH according to the experimental design. IVM was conducted by the following steps: Oocytes were cultured for 24 h in TCM 199 with 50 IU/L FSH (treatment, 800 COCs) and without FSH (control, 700 COCs), and placed in an incubator at 39°C under 5% CO_2_ with humidified air.

### Oocytes fixation and nuclear staining

After 24 h of incubation, first, matured oocytes were evaluated its morphology based on the expansion of cumulus cells and PB1 extrusion. Next, approximately 600 oocytes were denuded (cumulus cells were separated from the oocyte) through micropipette manipulation and enzymatic treatment. The oocytes were then stained with 1% aceto-orcein to observe the maturation rate [13]. Oocyte maturation was determined according to the changes in the chromosome configuration and nuclear membrane. The germinal vesicle (GV) was marked with nuclear membrane and the nucleus was distinctly visible on the edges. Germinal vesicle breakdown (GVBD) was marked with nuclear membrane rupture and the nucleolus not being distinctly visible. Metaphase I (MI) was indicated with homologous chromosome pairing and lined in the equator, anaphase I was identified by the centromere approaching the opposite pole and pulling homologous chromosomes into two parts, telophase I was marked with homologous chromosomes that were completely captured on each pole and MII was indicated by the extrusion of PB1 and the sequence of chromosome that was similar to MI.

### IVF and culture

To prove the ability of mature oocytes to develop into embryos, matured oocytes were processed for IVF. Four hundred matured oocytes of control group and 500 matured oocytes of treatment group continued for IVF. Oocytes were transferred into a petri dish containing fertilization medium and washed twice in IVF medium. Thawed semen was washed twice by centrifugation at 320× *g* for 5 min, in a non-capacitating HEPES-buffer (NC) sperm washing medium. The supernatant was removed, and sperm pellet was homogenized by pipetting in the remaining NC sperm wash medium to adjust sperm concentration and motility. A final concentration 2.0×10^6^ sperm/mL was added to the droplets, each 50-μL droplet containing five oocytes. Oocytes and sperms were incubated in Tyrode’s albumin lactate pyruvate for 24 h and placed in an incubator at 39°C under 5% CO_2_ with humidified air. The fertilization of oocytes was evaluated by examination of cleavage rate. After 24 h of sperm oocytes, the presumptive zygotes were removed from IVF culture droplets, then denuding by repeated pipetting in *in vitro* culture (IVC) washing medium supplemented with 0.2% hyaluronidase. Denuding presumptive zygotes were washed twice with IVC washing medium, and cultured in IVC medium (modified synthetic oviductal fluid) supplemented with 0.3% BSA was continued for 9 days.

### Statistical analysis

The results are expressed as mean±standard error of the mean. p<0.05 is considered statistically significant. The data were analyzed using one-way analysis of variance with a complete randomized design.

## Results

The results showed that FSH supplementation significantly increased oocyte maturation rate (69.21±7.261% vs. 43.25±6.23%). Furthermore, the rate of degenerated oocytes was lower in the medium with FSH supplementation (3.21±0.25% vs. 10.15±3.1%) (Table-1). From the 900 matured oocytes used in this experiment, it was revealed that embryo development post-IVF was higher in the FSH-supplemented group than in the control (without FSH supplementation). The number of embryos produced in the blastocyst stage was 12.43±2.15% and 22.28±4.86% (Table-2) for the control and the FSH-supplemented group, respectively.

**Table-1:** Effect of FSH supplementation on Bligon goat oocyte maturation following 24 h of incubation and arceto orcein staining (%).

FSH concentration	Number of oocytes	Meiosis development (%)			

		GV	GVBD	MII	Degenerated
Control	300	7.61±1.52	23.91±2.44	43.25±6.23^a^	10.17±3.15^a^
50 IU/L	300	7.02±4.51	18.86±4.07	65.21±7.26^b^	3.21±0.25^b^

FSH=Follicle-stimulating hormone, GV=Germinal vesicle, GVBD=Germinal vesicle break down, MII=Metaphase II. ^a,b^Means with different superscripts within a column differ significantly (p<0.05)

**Table-2:** Embryonic developmental stages of oocytes submitted to different FSH supplementation on Bligon goat oocyte following *in vitro* culture.

Treatment	Number of matured oocytes	Cleavage (%, Day 2)	Morula (%, Day 6)	Blastocyst (%, Day 9)
Control	400	56.65±3.91^a^	30.47±5.42^a^	12.43±2.15^a^
50 IU/L	500	71.21±3.29^b^	40.86±4.16^b^	22.28±4.86^b^

^a,b^Means with different superscripts within a column differ significantly (p<0.05). Day 0 is the fertilization day

## Discussion

FSH supplementation into IVM medium was effective in stimulating the development of oocytes. More MII oocytes were obtained from the FSH group than from the control group (p<0.05). Mature oocytes were characterized by the expansion of cumulus cells, GVBD, and the PB1 extrusion [12]. Expansion of cumulus cells was critical for successful fertilization as it aids the migration of spermatozoa between cumulus cells [3]. The addition of FSH in the maturation medium intensified the spread of cumulus cells around the oocyte, indicating that it increased the sperm capacity and the fertilization process.

FSH has a stimulatory effect on nuclear and cytoplasmic maturation of porcine oocytes. The addition of FSH in the first 20 h of culture increases cleavage and blastocyst development rates [8]. FSH supplementation to the culture medium significantly increased the cleavage rate in developed buffalo embryos up to the blastocyst stage, compared with the control medium [9]. The previous studies have shown that the supplementation of gonadotropins and FSH in the maturation media enhanced cytoplasmic maturation and fertilization ability of oocytes *in vitro* [8,14]. The higher concentration of gonadotropins such as FSH and luteinizing hormone (LH) in the culture medium increased the percentage of oocytes achieving MII, normal chromosomal alignment, normal configuration of the spindle, cortical granule migration, and mitochondrial aggregation [15]. The growth of oocytes is strongly influenced by the activity of FSH and LH [16,17]. The absence of hormones prevents the oocytes from further developing and causes disintegration. An optimum concentration of FSH (5 μg/mL) and LH (50 IU/mL) in IVM medium improved yak (*Bos grunniens*) oocyte competence for development post-IVF [18]. The cleavage and development of oocytes to blastocysts were improved in the IVM medium with additional hormones (FSH, LH, and E2) compared to hormone absence in oocyte maturation [19]. Furthermore, IVM medium supplemented with FCS, hormone gonadotropin (FSH and LH), and steroid (estradiol [E2]) resulted in cumulus cell maturation of yak oocytes after 23-24 h. This indicates that FSH, LH, and E2 play important roles in oocyte maturation and that these hormones are necessary during IVM of yak oocytes for subsequent development. Concurrently, only recombinant FSH injection resulted in a higher number of oocytes. In addition, the combination with LH in treatments resulted in similar number of embryos accessible for embryo transfer [20].

Another report showed that gonadotropin was extensively used in *in vitro* oocyte maturation [21]. It was reported that the addition of varying gonadotropin concentrations (7.5 IU/mL and 75 IU/mL) did not increase the maturation rate of bovine oocytes, but was nonetheless required for maturation. In human clinical practice, gonadotropin is also employed in IVM at a dose of 0.5 IU/mL human chorionic gonadotrophin (HCG) combined with 75 IU/mL FSH in TCM-199 supplemented with 20% FBS. This combination improved the maturation rate compared to the media lacking HCG [22]. The effect of FSH, LH, and HCG on porcine oocytes and found that they significantly and dose-dependently increased cytoplasmic and nuclear maturation [15]. FSH is crucial for oocyte competence to reach meiosis, undergo fertilization, and further embryonic development [23].

## Conclusion

FSH supplementation is effective at improving oocyte maturation *in vitro* and yields mature oocytes for future IVF. In addition, loss of oocyte developmental competence may be corrected by improving culture condition *in vitro*.

## Authors’ Contributions

DTW conceived the study design, conducted the fieldwork, performed the statistical analysis, and drafted the manuscript. MP conducted data interpretation and drafted the manuscript. All authors read and approved the final manuscript.
